# Influence of pelvic incidence–lumbar lordosis mismatch on surgical outcomes of total hip arthroplasty: a retrospective cohort study

**DOI:** 10.1007/s00590-025-04383-5

**Published:** 2025-06-17

**Authors:** Hisatoshi Ishikura, Toru Nishiwaki, Maaya Kudo, Shunsuke Minoji, Takeyuki Tanaka, Sakae Tanaka

**Affiliations:** 1 Shizuoka Red Cross Hospital, Shizuoka, Japan; 2 The University of Tokyo, Tokyo, Japan

**Keywords:** Total hip arthroplasty, Spinopelvic alignment, PI-LL mismatch, Hip-spine syndrome, Postoperative outcome

## Abstract

**Purpose:**

Pelvic incidence–lumbar lordosis (PI-LL) mismatch has been widely studied in spinal disorders; however, its impact on total hip arthroplasty (THA) outcomes remains unclear. This study evaluated the impact of preoperative PI-LL mismatch on postoperative functional recovery and spinopelvic dynamics in patients undergoing THA.

**Methods:**

This retrospective cohort study included 167 patients who underwent primary unilateral THA. Patients were categorised into two groups based on a PI-LL mismatch threshold of 10°: mismatch group (PI-LL ≥ 10°) and matched group (PI-LL < 10°). Preoperative characteristics, spinopelvic parameters, and clinical outcomes were analysed. The Numerical Rating Scale (NRS) for pain, modified Harris Hip Score (mHHS), and Western Ontario and McMaster Universities Osteoarthritis Index (WOMAC) were used to assess the hip condition preoperatively and 6 months postoperatively.

**Results:**

The mismatch group exhibited a significantly higher pelvic tilt (PT) and lower sacral slope (SS) than the matched group. Furthermore, the mismatch group demonstrated reduced spinal flexibility, as indicated by a significantly smaller difference in spinopelvic angle between maximum lateral flexion positions. The spinopelvic parameters (PI, LL, PT, and SS) remained stable from preoperative to postoperative assessments. Preoperative mHHS and WOMAC scores were significantly worse in the mismatch group. However, no significant differences in postoperative outcomes were observed between the groups at 6 months.

**Conclusion:**

PI-LL mismatch was associated with altered spinopelvic parameters and poorer preoperative functional scores. However, short-term outcomes after THA remained comparable. These findings underscore the importance of individualised preoperative assessment while supporting the efficacy of THA, irrespective of spinopelvic alignment.

## Introduction

Total hip arthroplasty (THA) is a widely performed surgical procedure that alleviates pain and restores function in patients with advanced hip osteoarthritis (OA) [1]. Although THA outcomes are generally favourable, the factors influencing postoperative recovery and functional improvement remain areas of ongoing investigation.

Spinopelvic alignment reflects the complex relationship between the spine and pelvis. This relationship, often referred to as the so-called “hip-spine syndrome”, holds significant importance in both spinal and hip surgical procedures [2, 3]. Recent studies have emphasised the growing recognition of spinopelvic alignment as a key determinant of surgical outcomes and patient satisfaction [3]. Optimal spinopelvic alignment supports an energy-efficient posture, promotes a balanced distribution of mechanical loads, and enhances overall spinal stability, ultimately contributing to better postoperative results [4]. In spinal surgery, preserving the appropriate spinopelvic alignment is essential for achieving solid spinal fusion, minimising the risk of adjacent segment degeneration, and optimising postoperative functional recovery [4]. The role of spinopelvic alignment in hip surgery has garnered attention because of its potential impact on hip mechanics and patient-reported outcomes [5].

Pelvic incidence (PI), pelvic kinematics, and sagittal balance provide a comprehensive understanding of hip-spine syndrome in individuals. Spinopelvic angular parameters characterise the structure and dynamics of the spinopelvic system [6, 7]. While PI is a fixed morphological parameter that offers insight into the physiological sagittal range of pelvic motion, other parameters such as sacral slope (SS), pelvic tilt (PT), and lumbar lordosis (LL) are functional and vary based on body posture.

Among these, the mismatch between the PI and LL (PI-LL mismatch) is a well-established parameter in spinal disorders, particularly in adult spinal deformities, where it is associated with poor clinical outcomes, such as back pain and reduced quality of life [8]. Specifically, the PI-LL mismatch is a known risk factor for adjacent segment degeneration in various types of spinal fusion surgery [9–12]. A study using biomechanical models indicated that the PI-LL mismatch tends to increase segmental axial loading in both unfused and fused lumbar spines [13].

The PI-LL mismatch may affect hip biomechanics by altering PT and acetabular orientation [14, 15]. Theoretically, these changes may influence the postoperative outcomes of THA. However, evidence regarding this topic remains sparse and inconclusive. This study aimed to clarify whether PI-LL mismatch affects the postoperative outcomes of THA. By addressing this gap, we hope to provide insights that can optimise surgical planning and patient management.

## Materials and methods

### Patients

**Fig. 1:**
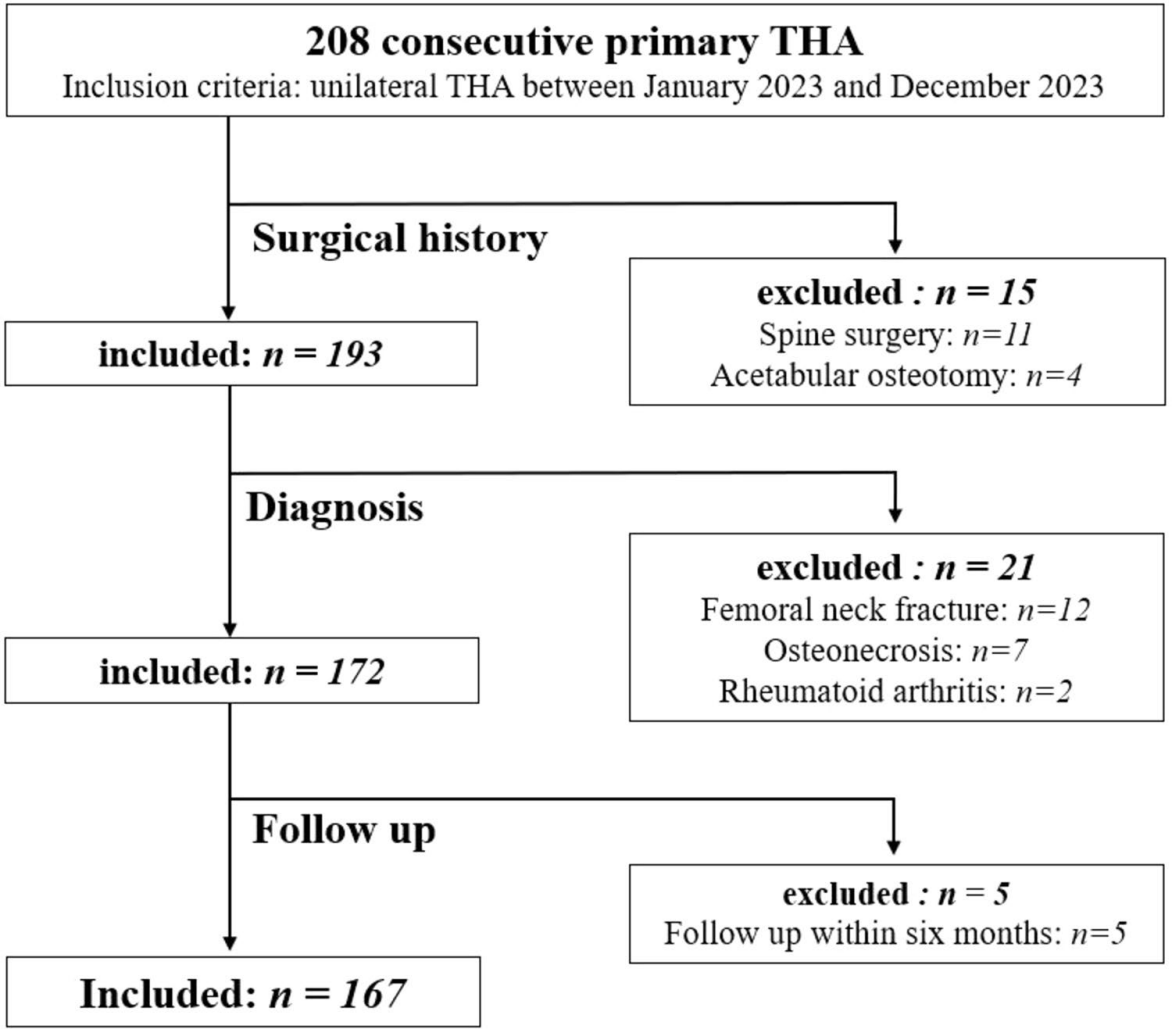
Patient flow chart. THA, total hip arthroplasty

This retrospective study enrolled 208 hips of 208 patients who underwent unilateral THA at our institution between January 2023 and December 2023. The exclusion criteria included a history of previous hip or spine surgery, diagnoses other than osteoarthritis, and patients with a follow-up period of < 6 months postoperatively. Therefore, 167 patients (167 hips) were included in this study (Fig. 1). The preoperative patient characteristics, including age, sex, height, body weight, body mass index (BMI), Kellgren–Lawrence grade of hip OA, and Crowe classification, were evaluated. Written informed consent was obtained from the patients or their family members.

### Radiographic measurements of spinopelvic parameters

**Fig. 2:**
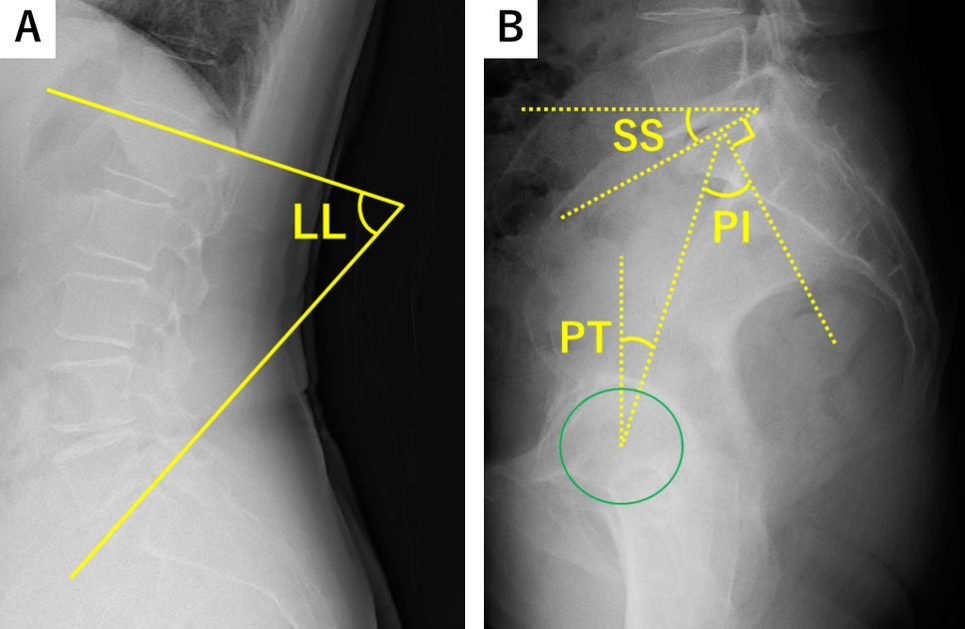
Methods of radiographic measurement of spinopelvic parameters. Methods for lumbar lordosis (LL), sacral slope (SS), pelvic tilt (PT), and pelvic incidence (PI) have been described. All the measurements were obtained using standing lateral plain radiographs. *LL* lumbar lordosis, *SS* sacral slope, *PT* pelvic tilt, *PI* pelvic incidence

Preoperative standing lateral spine radiographs were used to measure the PI, LL, PT, and SS (Fig. 2a, b). The PI-LL match or mismatch was determined based on the PI minus LL value, with a cut-off value of 10: mismatch group (≥ 10°) and matched group (< 10°). These spinopelvic parameters were also measured 6 months postoperatively.

**Fig. 3:**
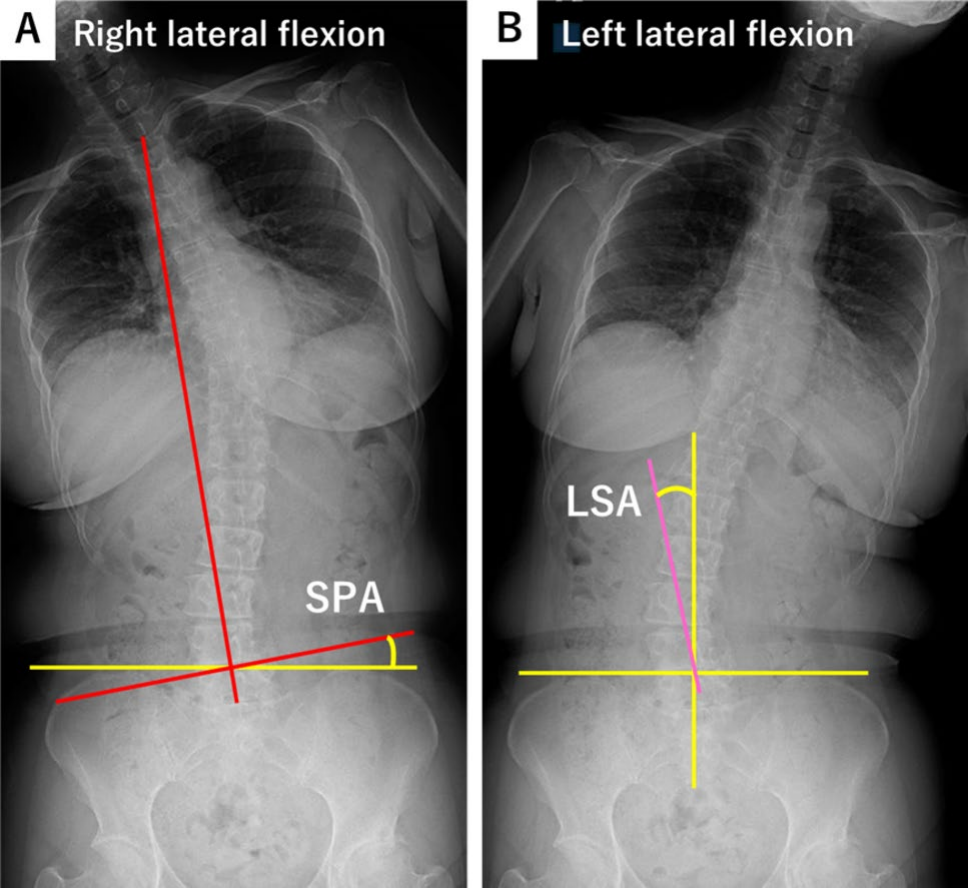
Standing whole-spine anteroposterior radiographs in right lateral bending and left lateral bending positions. Methods for measuring spinopelvic angle (SPA) and lumbosacral angle (LSA) were demonstrated. The SPA is the angle between a line drawn perpendicular to the middle of T1 to S1 (red) and the iliac crest line (yellow). The LSA is the angle between a line drawn through the spinous processes of L4 and L5 (pink) and is perpendicular to the iliac crest line (yellow). ΔSPA was defined as the absolute difference in SPA between maximum right and left lateral flexion positions. Similarly, LSA was defined as the absolute difference in the LSA between the maximum right and left lateral flexion positions. *SPA* spinopelvic angle, *LSA* lumbosacral angle

Spinopelvic angle (SPA) and lumbosacral angle (LSA) were assessed using preoperative standing anteroposterior spine radiographs, including the pelvis (Fig. 3a, b) [16].

The absolute difference in SPA between maximum right and left lateral flexion positions was defined as ΔSPA and used as an indicator of overall spinal flexibility. Similarly, the absolute difference in LSA between maximum right and left lateral flexion positions was defined as ΔLSA and used to assess lower lumbar flexibility.

### Surgical procedures, rehabilitation, and postoperative outcomes

All surgical procedures were performed using a minimally invasive anterior approach, with the assistance of a leg positioner [17]. This technique involves a small incision at the anterior aspect of the hip to preserve the intermuscular and neurovascular structures. Cup positioning was guided intraoperatively by fluoroscopy and a mechanical guide attached to the cup impactor, with a target orientation of 40° of abduction and 15° of anteversion based on the functional pelvic plane. For femoral stem alignment, preoperative three-dimensional planning based on CT scans was utilised to determine the optimal implantation position and orientation. However, no CT-based navigation systems, robotic assistance, or other computer-assisted technologies were used during the actual implantation process.

Postoperative rehabilitation commenced the day after surgery, with full weight-bearing ambulation permitted as soon as possible. Regarding range of motion, we did not impose any specific restrictions on hip flexion or extension within the limits of activities of daily living. During hospitalisation, the patients underwent training in activities of daily living, including stair climbing, and were discharged approximately 7–10 days postoperatively. During the first postoperative month, patients were instructed to sit with hips above knee level, considering their postoperative muscle strength and functional status. After one month, no specific limitations were applied, and patients were allowed to perform activities such as sitting on the floor and standing up from the floor as tolerated.

Clinical outcomes were evaluated using the Numerical Rating Scale (NRS) for pain, assessed separately at rest and during activity; the Western Ontario and McMaster Universities Osteoarthritis Index (WOMAC); and the modified Harris Hip Score (mHHS), preoperatively and 6 months postoperatively.

### Statistical analyses

Student’s t test was used to compare the mean values between the two groups. Differences in categorical variables were evaluated using the chi-squared test. Analyses were performed using Bell Curve for Excel ver. 4.04 (Social Survey Research Information Co. Ltd. Tokyo, Japan). All statistical analyses were performed at a significance level of *P* < 0.05.

## Results

### Patient demographics and surgical data

For all evaluated parameters, inter-observer intraclass correlation coefficients were greater than 0.9 (Table 1).

**Table 1:** Inter-observer Intraclass Correlation Coefficients for Each Parameter

	PI	LL	PT	SS	ΔLSA	ΔSPA
O1–O2	0.94	0.96	0.93	0.97	0.91	0.94
O1–O3	0.92	0.96	0.94	0.96	0.93	0.95
O2–O3	0.94	0.95	0.93	0.97	0.93	0.93

*O1* observer 1, *O2* observer 2, *O3* observer 3, *PI* pelvic incidence, *LL* lumbar lordosis, *PT* pelvic tilt, *SS* sacral slope, *LSA* lumbosacral angle, *SPA* spinopelvic angle

Among the 167 patients, 85 were classified into the mismatch group (PI-LL ≥ 10°) and 82 into the matched group (PI-LL < 10°). The mismatch group was significantly older (*p* = 0.007), but there were no significant differences between groups in terms of sex, height, weight, BMI, osteoarthritis grade, or Crowe classification. Additionally, operative time and intraoperative blood loss did not differ significantly between the two groups (Table 2).

**Table 2:** Patient demographics and surgical data

	PI-LL < 10 (n = 82)	PI-LL > 10 (n = 85)	P value
Age	67.6 ± 9.8	71.6 ± 9.2	0.007
Female (%)	72 (88%)	75 (88%)	0.932
Height (cm)	154.4 ± 6.8	153.6 ± 7.7	0.467
Weight (kg)	57.8 ± 11.9	56.2 ± 12.3	0.402
BMI (kg/m2)	24.1 ± 4.2	23.7 ± 4.5	0.558
*Hip OA grade*			
K-L 3 (%)	27 (33%)	29 (34%)	0.871
K-L 4 (%)	55 (67%)	56 (66%)	0.871
*Crowe type*			
1–2 (%)	77 (94%)	81 (95%)	0.691
3–4 (%)	5 (6%)	4 (5%)	0.691
Operation time (min)	76.7 ± 21.6	71.3 ± 17,4	0.117
Blood loss (mL)	164 ± 125	146 ± 130	0.235

*BMI* body mass index, *OA* osteoarthritis, *K-L* kellgren–lawrence, *PI* pelvic incidence, *LL* lumbar lordosis

### Spinopelvic parameters

Regarding the spinopelvic parameters, PI and PT were significantly higher, whereas LL and SS were significantly lower in the mismatch group (all *p* < 0.001). At 6 months postoperatively, no significant differences were observed in the PI, PT, LL, and SS compared with the preoperative values. The trends observed preoperatively, including significantly higher PI and PT and significantly lower LL and SS in the mismatch group, were consistent postoperatively (Table 2). Regarding spinal flexibility, no significant difference was observed in LSA between the groups; however, SPA was significantly lower in the mismatch group (Table 3).

**Table 3:** Spinopelvic parameters before and after total hip arthroplasty

	before THA			after THA		
	PI-LL < 10	PI-LL > 10	p value	PI-LL < 10	PI-LL > 10	p value
PI-LL	-0.2 ± 6.9	26.1 ± 15.3	< 0.001*	1.3 ± 9.8	23.2 ± 16.3	< 0.001*
PI	48.9 ± 10.2	57.5 ± 12.4	< 0.001*	47.9 ± 9.8	57.1 ± 12.7	< 0.001*
LL	49.1 ± 10.6	31.8 ± 19.7	< 0.001*	46.7 ± 11.4	33.9 ± 18.7	< 0.001*
PT	11.1 ± 6.5	26.5 ± 9.9	< 0.001*	12.1 ± 7.4	26.3 ± 11.2	< 0.001*
SS	37.7 ± 10.1	30.9 ± 12.7	< 0.001*	35.8 ± 9.1	30.8 ± 13.5	0.010*
ΔLSA	4.8 ± 9.8	3.9 ± 3.9	0.205	N/A	N/A	N/A
ΔSPA	20.8 ± 12.5	18.1 ± 8.9	0.023*	N/A	N/A	N/A

*THA* total hip arthroplasty, *PI* pelvic incidence, *LL* lumbar lordosis, *PT* pelvic tilt, *SS* sacral slope, *LSA* lumbosacral angle, *SPA* spinopelvic angle, *N/A* not applicable

*p < 0.05

### Clinical scores before THA

Preoperatively, the mismatch group had significantly worse modified Harris Hip Score (mHHS) results, particularly in subcategories such as limp, walking distance, and stair climbing (all *p* < 0.05). The WOMAC total score and function subscale were also significantly worse in the mismatch group (*p* = 0.039 and *p* = 0.011, respectively), suggesting greater functional impairment before surgery (Table 4).

**Table 4:** Pain and clinical scores before and after total hip arthroplasty

	Before THA			After THA		
	PI-LL < 10	PI-LL > 10	p value	PI-LL < 10	PI-LL > 10	p value
NRS_rest	3.2 ± 2.6	3.3 ± 2.7	0.858	0.2 ± 0.5	0.4 ± 1.0	0.131
NRS_activity	6.1 ± 2.4	6.0 ± 2.5	0.751	0.9 ± 1.2	0.8 ± 1.3	0.589
mHHS						
Total	56.5 ± 11.4	51.4 ± 14.0	0.021*	94.1 ± 5.3	93.9 ± 6.2	0.811
Pain	16.5 ± 7.0	14.5 ± 8.5	0.140	39.9 ± 2.3	39.8 ± 3.5	0.852
Limp	6.2 ± 1.5	5.7 ± 1.4	0.038*	10.8 ± 0.7	10.9 ± 0.4	0.295
Support	10.9 ± 0.7	10.6 ± 1.5	0.153	10.8 ± 0.9	10.7 ± 1.0	0.737
Distance walked	8.0 ± 3.0	6.6 ± 3.5	0.012*	10.8 ± 1.0	10.7 ± 0.9	0.797
Stairs	2.1 ± 0.7	1.8 ± 0.7	0.013*	3.8 ± 0.5	3.7 ± 0.8	0.141
Shoes and socks	2.1 ± 0.5	2.1 ± 0.5	0.966	3.5 ± 0.9	3.6 ± 0.8	0.741
Sitting	4.7 ± 0.7	4.6 ± 0.9	0.807	5.0 ± 0.0	5.0 ± 0.0	N/A
Public transport	1.0 ± 0.0	1.0 ± 0.1	0.305	1.0 ± 0.0	1.0 ± 0.1	0.305
WOMAC						
Total	39.3 ± 17.5	45.1 ± 16.4	0.039*	10.6 ± 8.9	10.3 ± 11.5	0.882
Pain	8.2 ± 3.6	8.9 ± 3.7	0.265	1.7 ± 1.8	1.7 ± 2.5	0.966
Stiffness	3.4 ± 2.0	3.1 ± 2.0	0.379	1.3 ± 1.4	0.9 ± 1.1	0.112
Function	27.8 ± 13.1	33.2 ± 12.8	0.011*	7.7 ± 6.6	7.7 ± 9.0	0.962

*THA* total hip arthroplasty, *NRS* numerical rating scale, *mHHS* modified harris hip score, *WOMAC* western ontario and McMaster Universities Osteoarthritis Index *N/A* not applicable

*p < 0.05

### Clinical outcomes at 6 months after THA

At 6 months after THA, no significant differences were observed between the two groups in NRS scores (at rest and during activity), mHHS, or WOMAC scores. Furthermore, there were no cases of dislocation or infection in either group during the follow-up period.

## Discussion

This study evaluated the impact of PI-LL mismatch on THA outcomes and found that while patients with PI-LL mismatch exhibited reduced spinopelvic flexibility and worse preoperative hip function, their postoperative recovery and functional outcomes were comparable to those without mismatch. These findings suggest that PI-LL mismatch may influence preoperative hip status more than postoperative recovery in patients undergoing THA.

Parameters such as LL, SS, and PT vary significantly depending on posture. In contrast, while some studies have reported minor changes in PI with postural shifts, it is generally considered a patient-specific, fixed anatomical parameter [7, 18]. Generally, PI itself is considered an indicator of spinopelvic flexibility. Patients with a high PI generally exhibit greater lumbar lordosis and increased spinopelvic mobility, allowing more posterior pelvic tilt during sitting. In contrast, individuals with a low PI tend to have decreased lumbar lordosis and limited posterior pelvic tilt, necessitating greater hip flexion during sitting. However, recent studies have suggested that PI-LL serves as a more sensitive indicator of spinopelvic imbalance [8]. Patients with a PI-LL mismatch tend to have relatively reduced LL and a more posteriorly tilted pelvis, even in the standing position, leading to a smaller SS and larger PT [12, 19]. Similarly, in this study, the mismatch group showed a significantly larger PT and smaller SS. The significantly smaller SPA observed in the PI-LL mismatch group suggests a correlation between reduced flexibility in the sagittal plane and diminished flexibility in the coronal plane of the spine. These findings are consistent with previous studies that reported PI-LL mismatch to be associated with altered sagittal alignment and reduced spinopelvic mobility. Buckland et al. demonstrated that patients with increased PI-LL mismatch exhibited limited postural pelvic mobility, which may affect functional acetabular positioning in total hip arthroplasty candidates [20]. Similarly, Schwab et al. found that PI-LL mismatch was significantly associated with spinal imbalance and disability in patients with adult spinal deformity [8]. These findings support the interpretation that PI-LL mismatch serves as a surrogate marker of spinopelvic stiffness, potentially influencing hip-spine mechanics even prior to surgical intervention.

This spinopelvic imbalance not only increases the load on the hip joint but also poses a potential risk of dislocation after THA [20]. Utilising a minimally invasive anterior approach in our study may have contributed to the lack of dislocations observed in either group, even in patients with a PI-LL mismatch. This technique preserves critical soft tissues and may offer greater stability than posterior approaches, as suggested in previous research [17]. Although this study did not modify the cup placement angles based on spinopelvic flexibility, techniques that involve adjusting cup orientation or selecting a dual mobility cup according to spinopelvic mobility are increasingly recognised as effective strategies to minimise the risk of impingement and dislocation [21]. Comparative studies investigating surgical approaches and implant positioning strategies in patients with PI-LL mismatch may provide additional insights into optimising surgical outcomes.

Several factors may explain why the PI-LL mismatch negatively affected the preoperative condition but did not influence the postoperative outcomes of THA. Generally, THA has minimal impact on sagittal alignment in static positions [22–25]. In this study, no significant differences were observed in various spinopelvic parameters between the preoperative and postoperative periods. However, a dramatic improvement in hip function after THA may alleviate spinal stress and minimise the effects of spinopelvic malalignment. Furthermore, in the PI-LL mismatch group, the coexistence of spinopelvic malalignment and hip osteoarthritis may have had a synergistic effect, exacerbating preoperative functional impairment. This relationship may have contributed to the deterioration of the preoperative clinical scores, including walking distance, stairs, and limp, as observed in this study.

The results of this study suggest that THA can dramatically improve patient status even in the presence of spinopelvic imbalance. However, it is important to consider the ceiling effect of patient-reported outcome measures (PROMs). For instance, previous studies have indicated that PROMs may not accurately reflect objectively assessed physical function, including gait speed and gait pattern [26, 27]. Further research, including gait analysis, is warranted to investigate how spinopelvic imbalance affects physical function.

This study had several limitations. First, this was a single-centre cohort study with a limited sample size. Although our study demonstrated no significant differences in short-term outcomes between the mismatch and matched groups, an expanded subgroup analysis with a larger sample size may provide additional insights. Second, measurements were performed manually using plain radiographs. Although measurement errors cannot be completely excluded when using manual radiographic assessment, all measurements were performed by three experienced orthopaedic surgeons, and the inter-observer ICCs exceeded 0.9 for all parameters, indicating strong reliability. Third, spinal symptoms such as low back pain or radiculopathy were not assessed in this study. Further research and validation are required to address these limitations.

Nevertheless, the strength of this study lies in its detailed investigation of the relationship between preoperative spinal alignment, particularly PI-LL mismatch, and postoperative outcomes after THA, making it a valuable contribution to the literature.

In conclusion, a PI-LL mismatch is associated with poorer preoperative function and reduced spinopelvic flexibility in patients undergoing THA. However, it did not significantly affect the short-term postoperative outcomes. These findings highlight the need for individualised preoperative assessment and suggest that THA remains an effective intervention regardless of spinopelvic alignment, indicating that the presence of a PI-LL mismatch should not be a reason to hesitate in performing THA.

## Data Availability

The datasets generated and analyzed during the current study are available from the corresponding author on reasonable request.
